# Scientific Evidence of Chinese Herbal Medicine (Gegen Qinlian Decoction) in the Treatment of Ulcerative Colitis

**DOI:** 10.1155/2022/7942845

**Published:** 2022-03-14

**Authors:** Jinke Huang, Jiaqi Zhang, Yifan Wang, Jing Ma, Xuefei Yang, Xiaoxue Guo, Mi Lv, Jinxin Ma, Yijun Zheng, Fengyun Wang, Xudong Tang

**Affiliations:** ^1^Department of Gastroenterology, Xiyuan Hospital of China Academy of Chinese Medical Sciences, Beijing, China; ^2^China Academy of Chinese Medical Sciences, Beijing, China; ^3^Department of Gastroenterology, Peking University Traditional Chinese Medicine Clinical Medical School (Xiyuan), Beijing, China; ^4^Graduate School, Beijing University of Chinese Medicine, Beijing, China

## Abstract

**Objectives:**

Gegen Qinlian decoction (GQD), a Chinese herbal compound, has been widely used in the treatment of ulcerative colitis (UC) in China. However, evidence from systematic reviews (SRs)/meta-analyses (MAs) of GQD in UC remains highly controversial. To collate, evaluate, and synthesize the current evidence, we carried out this study.

**Methods:**

SRs/MAs of GQD for UC were obtained from eight databases. Methodological Quality of Systematic Reviews 2 (AMSTAR-2) was utilized to appraise the methodological quality, Preferred Reporting Item for Systematic Reviews and Meta-Analyses (PRISMA) for reporting quality, and Grading of Recommendations Assessment, Development, and Evaluation (GRADE) for evidence quality.

**Results:**

Four eligible SRs/MAs were obtained. According to AMSTAR 2, all SRs/MAs were graded as critically low quality. According to PRISMA checklist, all SRs/MAs failed to report the information of protocol and registration. With GRADE, no outcome measure with high-quality evidence was found, and the evidence quality for outcome measures was in the moderate to critically low levels.

**Conclusions:**

GQD with conventional medicine (CM) seems to be more effective in UC than CM alone. This finding provides a new alternative strategy for the treatment of UC. However, owing to the limitations of the evidence provided by the included SRs/MAs, this conclusion must be treated with caution.

## 1. Introduction

Ulcerative colitis (UC), a major form of inflammatory bowel disease, is characterized by remitting and relapsing mucosal inflammation that begins in the rectum and extends into the colon [1]. Abdominal pain and uncontrolled diarrhea mixed with blood are the main symptoms of UC, and the long-term maintenance of these symptoms causes serious distress to patients [2]. The incidence and prevalence of UC are steadily increasing, with 38 per 100,000 individuals per year in the United States [3] and 35 to 50 per 100,000 inhabitants in Northern Europe [4]. The mechanisms underlying UC are not fully defined; there is increasing evidence that environmental influences, microbiome imbalances, genetic variation, and disturbances in innate and adaptive immune responses are all associated with UC [5]. Although great progress has been made in the treatment of UC, there is still no single ideal therapy [6]. Most new drugs and action protocols could only control part of the UC symptoms with low efficacy [7]. Therefore, the search for effective therapeutic strategies is urgently needed.

As a Chinese herbal compound, Gegen Qinlian decoction (GQD) has been widely used in clinical treatment of UC. GQD contains a variety of effective active ingredients, such as berberine, baicalin, and puerarin [8]. Accumulating evidence suggests that these components are effective in improving UC symptoms in animal models [9]. Based on the theory of evidence-based medicine, systematic reviews (SRs)/meta-analyses (MAs) are considered the gold standard to appraise the benefits of clinical interventions. The initial search revealed several SRs/MAs on the treatment of UC with GQD that have been published. However, their quality varies and the results are highly controversial, which limits the use of evidence. Hence, to collate, evaluate, and integrate the results from these SRs/MAs, we performed this overview [10].

## 2. Methods

The method used for this overview follows the Cochrane Handbook, and the protocol has been registered on PROSPERO (CRD42021273358).

### 2.1. Search Strategy

SRs/MAs of GQD for UC were obtained from Cochrane Library, PubMed, Web of Science, Embase, China National Knowledge Infrastructure, Chinese Scientific Journal Database, Wanfang databases, and Chongqing VIP. Search period was from database establishment to October 2021. Ulcerative colitis, Chinese Medicine, Gegen Qinlian decoction, and systematic review were used as search keywords.  Table 1 presents a search strategy for PubMed.

### 2.2. Inclusion and Exclusion Criteria

SRs/MAs that conformed to the following criteria were involved: (1) participants: individuals diagnosed with UC according to appropriate diagnostic criteria; (2) type of design: SRs/MAs only enrolled randomized controlled trials; (3) intervention: GQD or in combination with conventional medication (CM) versus CM; (4) outcomes: effective rate, recurrence rate, level of serum inflammatory factor, ulcerative colitis endoscopic index of severity (UCEIS), and adverse events.

### 2.3. Data Extraction

Data extraction was performed by two independent reviewers. Literature screening was performed by two parts. Titles and abstracts were read for primary screening firstly, and full texts of initially eligible articles were further read to identify the final articles. The following items were included in the data extraction: (1) general information, (2) characteristics (sample size, intervention), and (3) results (outcomes, relative effect). Any disagreements were resolved by an experienced third reviewer.

### 2.4. Quality Assessment

Quality assessment was performed by two independent reviewers, and any disagreements were resolved by an experienced third reviewer. Methodological Quality of Systematic Reviews 2 (AMSTAR-2) [11] was utilized to appraise the methodological quality, Preferred Reporting Item for Systematic Reviews and Meta-Analyses (PRISMA) [12] for reporting quality, and Grading of Recommendations Assessment, Development, and Evaluation (GRADE) [13] for evidence quality.

## 3. Results

### 3.1. Literature Screening

**Figure 1:**
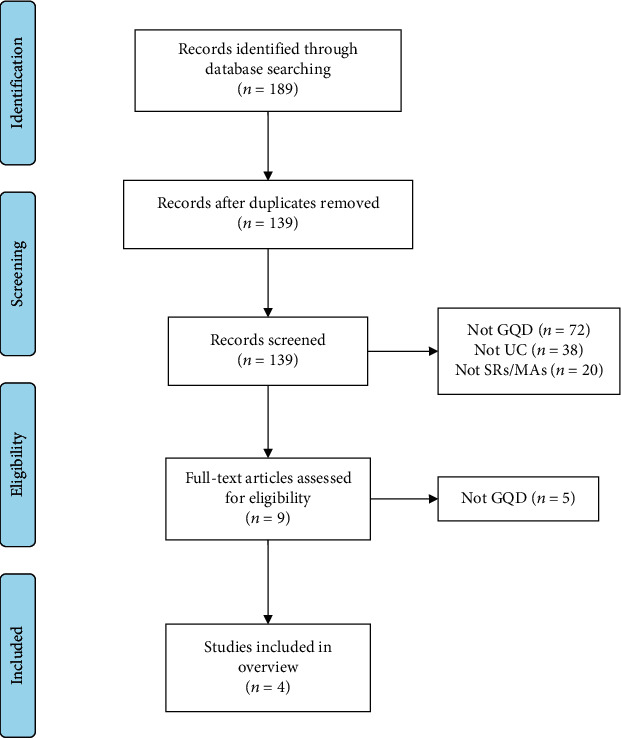
A flowchart of the literature selection process.

As shown in Figure 1, 189 citations were obtained from the initial searches, and 50 duplicates were removed. In the first step of screening, 139 irrelevant citations were excluded by reading the titles and abstracts; in the second step of screening, 5 irrelevant citations were excluded by reading the full text. Ultimately, the remaining 4 articles [14–17] met the inclusion criteria for this study.

### 3.2. Study Characteristics

All included studies were conducted in China and published in recent years (2019-2021). Simple size ranged from 381 to 2028. GQD plus CM was applied as experimental intervention, while CM alone was applied as the control intervention in all studies. All reviews applied the Cochrane criteria tool for methodological quality assessment of included trails. Further details are presented in Table 2.

### 3.3. Quality Assessment of the Included Studies

#### 3.3.1. Methodological Quality

According to the results of AMSTAR-2, all included studies failed to meet the entry requirements and were therefore rated as critically low methodological quality. The main defects were concentrated in item 2 (no study provided protocol and registration), item 4 (only one study provided the search strategy), and item 7 (a list of excluded trails was missing in all studies). Further details are shown in Table 3.

#### 3.3.2. Reporting Quality

According to the results of PRISMA, title, abstract, introductions, results, discussion, and funding were completely reported in all studies. However, in Methods, information of protocol and registration was missing in all studies. Furthermore, information of search strategy was missing 75% of the included studies. Further details are shown in Table 4.

#### 3.3.3. Evidence Quality

According to GRADE, 12 outcome indicators were appraised, of which 5 were of moderate quality, 5 were of low quality, and 2 were of critical low quality. The factors affecting the evidence quality were risk of bias, imprecision, publication bias, and inconsistency. Further details are shown in Table 5.

### 3.4. Descriptive Analysis

#### 3.4.1. Description of Efficacy

Relative effects associated with efficacy of GQD in UC are shown in Table 5. Effective rate was utilized in three studies [14, 16, 17] to evaluate the effect of GQD for UC; the pooled results suggested that the GQD group was super to CM. Recurrence rate was utilized in two studies [14, 17]; the pooled results suggested that the GQD group was super to CM. Levels of TNF-*α* and IL-6 were reported in one review [15]; results revealed that GQD plus CM had an advantage over the CM group. One review [16] compared mucosal improvement in the GQD and CM groups; the results suggested no statistical difference between these two groups. One review [17] reported the results of the UCEIS score; the pooled results suggested that the GQD group was super to the CM group.

#### 3.4.2. Description of Safety

Three reviews [14, 16, 17] reported on the outcome of adverse events. Two of which [14, 17] showed that the use of GQD in combination with CM reduced the incidence of adverse events, while the other [16] showed no statistical difference in the incidence of adverse events between the combination group and the CM group, which may be attributed to the small sample size.

## 4. Discussion

SRs/MAs are considered the gold standard for evaluating health interventions. However, evidence provided by high-quality SRs/MAs is credible, while low-quality evidence may mislead clinical decisions [18]. Thus, there is a gap between the use of evidence and its practical implementation in real-world dynamics. In response to this issue, the method of overview of SRs/MA is brought up by evidence-based medicine experts [19], with the purposes of evaluation and synthesis of evidence on the same topic [19]. In China, GQD has been widely used for the clinical treatment of UC. However, the published SRs/MAs emphasize that this therapy is still not fully implemented in a real-world context. To collate, appraise, and synthesize the current evidence, we therefore carried out this study.

In this study, methodological quality, reporting quality, and evidence quality of the included reviews were appraised. Our results suggest that the use of GQD in combination with CM is beneficial in patients with UC, with improved effective rate and UCEIS scores and reduced relapse rates, serum inflammatory markers, and adverse events. However, these findings must be considered cautiously owing to the limitations of the enrolled reviews. Notably, almost all included SRs/MAs suggested that GQD plus CM appeared to have a significant benefit in the treatment of UC; nevertheless, most authors did not wish to draw firm conclusions owing to the small sample size or low methodological quality of the included trials. With AMSTAR-2 results, neither I2 (protocol and registration) nor I7 (list of excluded trials) were followed, which is likely to increase the risk of bias and weaken the reliability of the results. With PRISMA results, information of I5 (protocol and registration) and I8 (search) was severely missing, which seriously undermines the rigor of SRs/MAs. For GRADE results, no high-quality evidence was found, suggesting that the results from the included reviews may differ from the real results and cannot provide reliable available evidence. Although quality from the included SRs/MAs is generally low and defects are frequent, this also means that there is much room for progress in the SR/MA process. Our study highlights areas of methodology that need to be improved, which have directionally guiding value for rapidly improving the quality of evidence in the future.

GQD consists of four Chinese herbal medicines, Radix Puerariae, licorice, Coptidis Rhizoma, and Scutellariae Radix. Based on traditional Chinese medicine theory, “dampness-heat” is the core of UC. Intestinal damp-heat can stimulate qi stagnation and blood stasis to damage the intestinal mucosa, so that patients will have diarrhea, pus, and bloody stools [20]. Therefore, the use of methods to remove damp-heat may contribute to the healing of the diseased intestinal mucosa [21]. In addition, experimental studies have also preliminarily revealed the pharmacological effects of GQD in the treatment of UC. It has been reported that GQD can inhibit Toll-like receptor 4/nuclear factor-kB signaling, which in turn relieves UC symptoms and repairs the intestinal epithelial barrier [22]. Moreover, GQD was observed to regulate Th17/Treg cell homeostasis by inhibiting IL-6/JAK2/STAT3 signaling in DSS-induced UC mice, which in turn alleviated symptoms [9]. Network pharmacology has also found that GQD can reduce the degree of inflammation in ulcerative colitis by downregulating the EGFR/PI3K/AKT signaling pathway and inhibiting the release of proinflammatory cytokines [23]. Baicalin, puerarin, baicalin, berberine, and glycyrrhizic acid, as the main components of GQD, have also been found to have antiviral and antidiarrheal effects and may be beneficial in improving the symptoms of UC [24]. Additionally, the combination of Radix Puerariae, Radix Glycyrrhizae, and Rhizoma Coptidis has been observed to drive the repair of colonic mucosa according to the internal meridian [25]. Given the current findings, the mechanism of GQD in UC involves multiple components and multiple targets and may be a promising therapeutic strategy.

This is the first study to evaluate and synthesize the evidence of GQD in combination with CM for UC, which may provide evidence reference for the treatment decision of UC. Moreover, our study highlights areas of methodology that need to be improved, which may help guide future high-quality SRs/MAs. Nevertheless, limitations should be acknowledged, as quality evaluation is based on subjective assessment tools, and the assessment results may vary from reviewer to reviewer.

## 5. Conclusion

GQD with conventional medicine (CM) seems to be more effective in UC than CM alone. This finding provides a new alternative strategy for the treatment of UC. However, owing to the limitations of the evidence provided by the included SRs/MAs, this conclusion must be treated with caution.

## Figures and Tables

**Table 1:** Search strategy for the PubMed database.

Query	Search term
#1	Ulcerative colitis [Mesh]
#2	Ulcerative colitis[Title/Abstract] OR idiopathic proctocolitis[Title/Abstract] OR ulcer colonitis[Title/Abstract] OR colitis gravis[Title/Abstract] OR inflammatory bowel disease[Title/Abstract]
#3	#1 OR #2
#4	Traditional Chinese Medicine[Mesh]
#5	Chinese Medicine[Title/Abstract] OR Gegen Qinlian[Title/Abstract] OR Gegen Qinlian decoction [Title/Abstract] OR herbal medicine[Title/Abstract]
#6	#4 OR #5
#7	Meta-analysis as Topic[Mesh]
#8	Systematic review[Title/Abstract] OR meta-analysis[Title/Abstract] OR meta analysis[Title/Abstract] OR meta-analyses OR metaanalysis[Title/Abstract]
#9	#7 OR #8
#10	#3 AND #6 AND #9

**Table 2:** Characteristics of the included reviews.

Author, year	Country	Sample	Treatment intervention	Control intervention	Quality assessment	Diagnostic criteria	Outcomes	Conclusion summary
Tang et al. [14], 2021	China	15 (1323)	GQD + CM	CM	Cochrane criteria	Consensus on the diagnosis and treatment specifications for inflammatory bowel disease in China (2007)	①, ②, ⑦	Compared with WMs, GQD or combined with sulfasalazine appeared to be more effective for UC. This finding provides a new therapeutic option for the treatment of UC. Nevertheless, due to the limitations of the included studies, further studies with higher quality and more rigorous design are needed to confirm the results of this study.
Xing [15], 2021	China	10 (861)	GQD + CM	CM	Cochrane criteria	Consensus on the diagnosis and treatment specifications for inflammatory bowel disease in China (2007)	⑤, ⑥	GQD combined with CM can more effectively improve the effective rate of patients with UC and improve the level of serum inflammatory factors. However, further implementation of larger and higher quality clinical trials is necessary to verify the conclusion.
Qin et al. [16], 2019	China	5 (381)	GQD + CM	CM	Cochrane criteria	Consensus on the treatment of ulcerative colitis in Chinese medicine (2010)	①, ③, ⑦	GQD combined with CM can more effectively improve the effective rate of patients with UC. Nevertheless, due to the limitations of this study, high-quality studies are still needed to further strengthen the conclusions of this study.
Fan [17], 2019	China	22 (2028)	GQD + CM	CM	Cochrane criteria	Third European evidence-based consensus on diagnosis and management of UC	①, ②, ④, ⑦	The combination of GQD with CM has potential benefits in the treatment of UC. However, due to the poor methodological quality of the included studies, there is insufficient evidence to draw firm conclusions to support the role of GQD for UC. Large-sample studies with more rigorous designs should be conducted to further establish clinical evidence.

①: effective rate; ②: recurrence rate; ③: mucosal improvement; ④: UCEIS score; ⑤: level of TNF-*α*; ⑥: level of IL-6; ⑦: adverse events.

**Table 3:** Result of the AMSTAR-2 assessments.

Reviews	AMSTAR-2																Quality
	I1	I2	I3	I4	I5	I6	I7	I8	I9	I10	I11	I12	I13	I14	I15	I16	
Tang et al. [14], 2021	Y	PY	Y	PY	Y	Y	N	Y	Y	Y	Y	Y	Y	Y	Y	Y	CL
Xing [15], 2021	Y	PY	Y	PY	Y	Y	N	Y	Y	Y	Y	Y	Y	Y	Y	Y	CL
Qin et al. [16], 2019	Y	PY	Y	PY	Y	Y	N	Y	Y	Y	Y	Y	Y	Y	Y	Y	CL
Fan [17], 2019	Y	PY	Y	Y	Y	Y	N	Y	Y	Y	Y	Y	Y	Y	Y	Y	CL

Y: yes; PY: partial yes; N: no; CL: critically low; L: low; H: high.

**Table 4:** Result of the PRISMA assessments.

Section/topic	Items	Tang et al., 2021	Xing, 2021	Qin et al., 2019	Fan, 2019	Compliance (%)
Title	(Q1) Title	Y	Y	Y	Y	100%

Abstract	(Q2) Structured summary	Y	Y	Y	Y	100%

Introduction	(Q3) Rationale	Y	Y	Y	Y	100%
	(Q4) Objectives	Y	Y	Y	Y	100%

Methods	(Q5) Protocol and registration	N	N	N	N	0%
	(Q6) Eligibility criteria	Y	Y	Y	Y	100%
	(Q7) Information sources	Y	Y	Y	Y	100%
	(Q8) Search	PY	PY	PY	Y	25%
	(Q9) Study selection	Y	Y	Y	Y	100%
	(Q10) Data collection process	Y	Y	Y	Y	100%
	(Q11) Data items	Y	Y	Y	Y	100%
	(Q12) Risk of bias in individual studies	Y	Y	Y	Y	100%
	(Q13) Summary measures	Y	Y	Y	Y	100%
	(Q14) Synthesis of results	Y	Y	Y	Y	100%
	(Q15) Risk of bias across studies	Y	Y	Y	Y	100%
	(Q16) Additional analyses	Y	Y	Y	Y	100%

Results	(Q17) Study selection	Y	Y	Y	Y	100%
	(Q18) Study characteristics	Y	Y	Y	Y	100%
	(Q19) Risk of bias within studies	Y	Y	Y	Y	100%
	(Q20) Results of individual studies	Y	Y	Y	Y	100%
	(Q21) Synthesis of results	Y	Y	Y	Y	100%
	(Q22) Risk of bias across studies	Y	Y	Y	Y	100%
	(Q23) Additional analysis	Y	Y	Y	Y	100%

Discussion	(Q24) Summary of evidence	Y	Y	Y	Y	100%
	(Q25) Limitations	Y	Y	Y	Y	100%
	(Q26) Conclusions	Y	Y	Y	Y	100%

Funding	(Q27) Funding	Y	Y	Y	Y	100%

Y: yes; PY: partial yes; N: no.

**Table 5:** Certainty of evidence quality.

Author, year	Outcomes	Limitations	Inconsistency	Indirectness	Imprecision	Publication bias	Relative effect (95% CI)	Quality
Tang et al. [14], 2021	Effective rate	-1	0	0	0	0	OR 3.77 (2.61, 5.45)	M
	Adverse events	-1	0	0	0	0	OR 0.43 (0.21, 0.90)	M
	Recurrence rate	-1	0	0	-1	0	OR 0.16 (0.05, 0.50)	L

Xing [15], 2021	Level of TNF-*α*	-1	0	0	-1	-1	SMD -0.81 (-1.07, -0.54)	CL
	Level of IL-6	-1	-1	0	-1	-1	SMD -1.20 (-2.00, -0.41)	CL

Qin et al. [16], 2019	Effective rate	-1	0	0	0	0	RR 1.18 (1.06, 1.30)	M
	Adverse events	-1	0	0	-1	0	RR O.11 (0.041, 1.92)	L
	Mucosal improvement	-1	0	0	-1	0	RR 1.13 (0.95, 1.88)	L

Fan [17], 2019	Effective rate	-1	0	0	0	0	RR 1.21 (1.16, 1.27)	M
	Recurrence rate	-1	0	0	-1	0	RR 0.18 (0.06, 0.61)	L
	Adverse events	-1	0	0	0	0	RR 0.37 (0.15, 0.90)	M
	UCEIS score	-1	-1	0	0	0	MD -0.63 (-1.26, -0.01)	L

## Data Availability

All analyses were based on previously published studies; thus, no data is required.

## Abbreviations

GQD: Gegen Qinlian decoction
UC: Ulcerative colitis (UC)
SR: Systematic review
MA: Meta-analysis
AMSTAR-2: Assessing the Methodological Quality of Systematic Reviews 2
UCEIS: Ulcerative colitis endoscopic index of severity
GRADE: Grading of Recommendations, Assessment, Development, and Evaluation
PRISMA: Preferred Reporting Item for Systematic Reviews and Meta-Analyses
CM: Conventional medicine.
